# The complete plastid genome of cherry plants *Prunus discoidea* (Rosaceae) and its phylogenetic implication

**DOI:** 10.1080/23802359.2019.1677519

**Published:** 2019-10-18

**Authors:** Meng Li, Yan-Feng Song, Shu-Xia Zhu, Hong Zhu, Xian-Gui Yi, Zhang-Chi Chen, Ming-Zhi Li, Xian-Rong Wang

**Affiliations:** a Co-Innovation Center for Sustainable Forestry in Southern China, Nanjing Forestry University, Nanjing, Jiangsu, China;; b Cherry Research Institute, College of Biology and the Environment, Nanjing Forestry University, Nanjing, Jiangsu, China;; c Nanjing Foreign Language School, Nanjing, Jiangsu, China;; d Genepioneer Biotechnologies Co. Ltd, Nanjing, Jiangsu, China

**Keywords:** Plastid, endemic flowering cherry, eastern China, *Prunus discoidea*, *Cerasus*

## Abstract

*Prunus discoidea* is an endemic cherry species with ornamental value, spread in eastern China (Anhui, Jiangxi, Zhejiang provinces). Little information is available regarding its genomic, with limited phylogenetic relationship study performed on *P. discoidea* until now. The plastid genome was 158,024 bp in length consisting of four regions: large single-copy region (85,953 bp), small single-copy region (19,113 bp), and a pair of inverted repeat regions (26,469 bp each). The plastid genome contained a total of 129 genes, including 84 coding genes, 8 rRNA genes, and 37 tRNA genes. Phylogenetic analysis for 20 reported genomes within the *Prunus sensu lato* showed three main clades of *Prunus s.l.* with strong supports.

*Prunus* L. *s.l.* containing ca. 400 species, is mainly distributed in the northern hemisphere (Huxley et al. 1999). Subgenus *Cerasus* comprising ca. 40 species are mainly distributed in eastern Asia, and includes fruit trees and garden ornamentals. *Prunus discoidea* (T.T. Yu & C.L. Li) Z. Wei & Y.B. Chang is an endemic cherry species of eastern China, belonging to Subgenus *Cerasus*. However, the genetic relationship of *P. discoidea* relative to other flowering cherries is poorly understood. Furthermore, the species is a valuable ornamental plant with various flower colours from white to pink. Here, we assembled the plastid genome of *P. discoidea* and showed its phylogenetic relationship in *Prunus s.l.* which will be useful for future studies on the breeding and conservation of flowering cherry.

The plant material was obtained from Huangshan, Anhui province, China (30°07′58.5″N 118°09′43.2″E, altitude 1200 m). The voucher specimen was deposited at Nanjing Forestry University (NF: 161093753). Total DNA was extracted from fresh leaves with a modified CTAB protocol. The whole genome sequencing was conducted by Nanjing Genepioneer Biotechnologies Inc. (Nanjing, China) on the Illumina Hiseq 2500 platform (Illumina, San Diego, CA). A total of 2.82 Gb clean PE reads (Phred scores >20) were assembled using the programme SPAdes assembler 3.10.0 (Bankevich et al. 2012). The plastome was annotated by Dual Organellar GenoMe Annotator (DOGMA) (Wyman et al. 2004).

The complete circular plastid genome of *P. discoidea* (GenBank accession MN158647) was 158,024 bp in length. Consisting of four regions; large single-copy region (LSC) of 85,953 bp, small single-copy region (SSC) of 19,113 bp, and a pair of inverted repeat regions (IRA and IRB) of 26,469 bp each. The overall GC contents of the plastid genome were 36.8%; LSC (34.7%), SSC (30.1%), and IR (42.4%). The genome contained a total of 129 genes, including 84 coding genes, 8 rRNA genes, and 37 tRNA genes.

The complete plastid genome sequence of other 20 *Prunus* species (*Neillia gracilis* AF487143 and *Neillia serratisepala* KY419969 as outgroups) were aligned using MAFFT (Katoh and Standley 2013). Maximum-likelihood (ML) analysis was conducted in FastTree v 2.1.10 (Price et al. 2010). The ML tree (Figure 1) showed that the genus *Prunus* was strongly supported by three main clades of *Prunus s.l.* (BS 100%) and *P. discoidea* was most closely related to *Prunus serrulata* var. *spontanea*.

**Figure 1.**
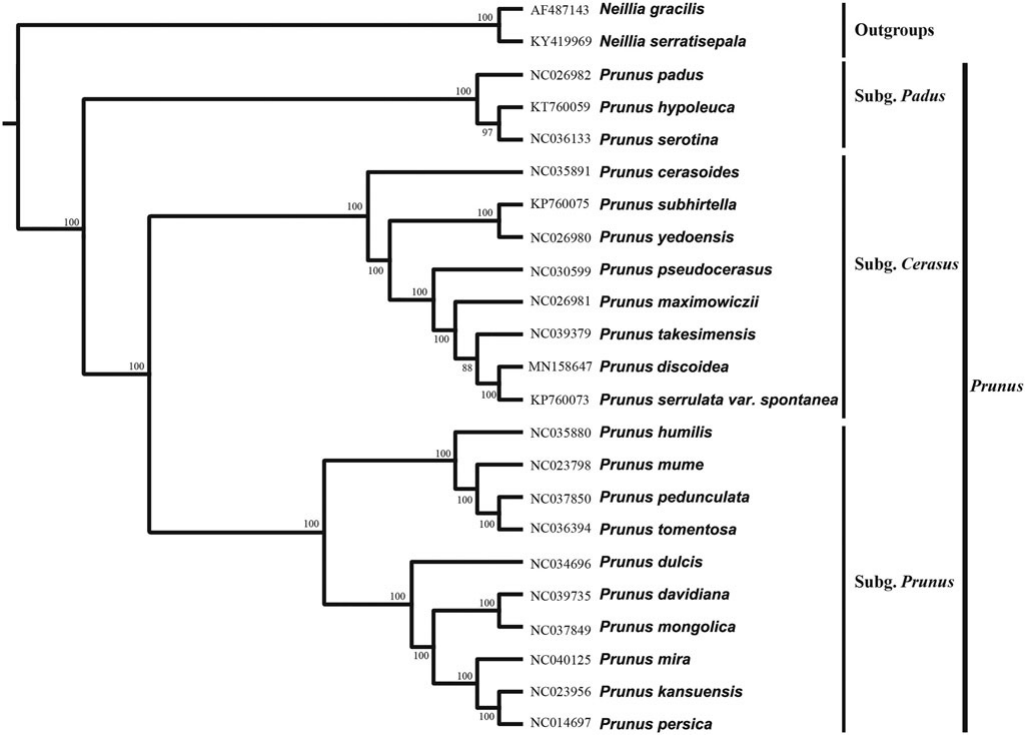
Maximum-likelihood phylogenetic tree for *Prunus discoidea* based on 23 complete plastid genomes. *Neillia gracilis* and *Neillia serratisepala* (Rosaceae) were used as outgroup and the support values are shown at the branches.
